# The effects of combination treatments on drug resistance in chronic myeloid leukaemia: an evaluation of the tyrosine kinase inhibitors axitinib and asciminib

**DOI:** 10.1186/s12885-020-06782-9

**Published:** 2020-05-07

**Authors:** H. Jonathan G. Lindström, Ran Friedman

**Affiliations:** Department of Chemistry and Biomedical Sciences, Linnæus University, Kalmar, 391 82 Sweden

**Keywords:** Allosteric inhibitor, Targeted therapy, Drug combination

## Abstract

**Background:**

Chronic myeloid leukaemia is in principle a treatable malignancy but drug resistance is lowering survival. Recent drug discoveries have opened up new options for drug combinations, which is a concept used in other areas for preventing drug resistance. Two of these are (I) Axitinib, which inhibits the T315I mutation of BCR-ABL1, a main source of drug resistance, and (II) Asciminib, which has been developed as an allosteric BCR-ABL1 inhibitor, targeting an entirely different binding site, and as such does not compete for binding with other drugs. These drugs offer new treatment options.

**Methods:**

We measured the proliferation of KCL-22 cells exposed to imatinib–dasatinib, imatinib–asciminib and dasatinib–asciminib combinations and calculated combination index graphs for each case. Moreover, using the median–effect equation we calculated how much axitinib can reduce the growth advantage of T315I mutant clones in combination with available drugs. In addition, we calculated how much the total drug burden could be reduced by combinations using asciminib and other drugs, and evaluated which mutations such combinations might be sensitive to.

**Results:**

Asciminib had synergistic interactions with imatinib or dasatinib in KCL-22 cells at high degrees of inhibition. Interestingly, some antagonism between asciminib and the other drugs was present at lower degrees on inhibition. Simulations revealed that asciminib may allow for dose reductions, and its complementary resistance profile could reduce the risk of mutation based resistance. Axitinib, however, had only a minor effect on T315I growth advantage.

**Conclusions:**

Given how asciminib combinations were synergistic in vitro, our modelling suggests that drug combinations involving asciminib should allow for lower total drug doses, and may result in a reduced spectrum of observed resistance mutations. On the other hand, a combination involving axitinib was not shown to be useful in countering drug resistance.

## Background

Targeted therapies have revolutionised the treatment of chronic myeloid leukaemia (CML) since their introduction. CML is driven by the fusion protein Bcr-Abl1, which results from a chromosomal translocation that makes the Abl1 tyrosine kinase constitutively active. Accordingly, treatment involves highly specific ATP-competitive tyrosine kinase inhibitors (TKIs) that bind to the ATP-binding pocket of Abl1, deactivating the enzyme. However, TKI treatment is rarely curative and therefore has to be continued for many years, if not indefinitely [1]. In many cases, patients eventually develop mutations in the Abl1 kinase domain which limits the effectivity of TKIs and consequently restores proliferative signalling leading to a return of the disease [2].

The most problematic Abl1 resistance mutation is T315I, the so-called gatekeeper mutation, which confers resistance to all approved TKIs except one: ponatinib. However, ponatinib is used sparingly due to side effects and concerns about toxicity. It has been suggested that T315I inhibits TKI binding primarily by sterically clashing with most drugs because of its location at the entrance of the ATP-binding pocket [3]. Other resistance mechanisms include kinetic stabilisation of a protein conformation that does not bind the drug [4], mutants that increase the catalytic activity of the drug target [5], and activation of other proteins than the drug target [6, 7].

All TKIs that are currently involved in CML therapy, including ponatinib, are less effective against T315I than against wildtype Bcr-Abl1 (the drug concentration required to halve the growth of Bcr-Abl1 positive cells, IC_50_, is higher for T315I mutants, see Table 1). This leads to an expansion of the T315I-positive sub-population of CML progenitor cells as it is favoured by evolution, even if disease symptoms are effectively suppressed [8]. Recently, it has been reported that axitinib, a vascular endothelial growth factor receptor TKI used in advanced renal cell carcinoma, is an effective inhibitor of Bcr-Abl1 T315I but not of native Bcr-Abl1 or of most other mutants. Axitinib cannot be used as a standalone treatment against cells that carry the native Bcr-Abl1 gene, since achievable plasma concentrations are too low for effective inhibition of the native protein [9–11]. This raises the question: Is it possible to combine axitinib with another TKI such that the treatment is both effective and evolutionarily selects against T315I? Such a combination could effectively prevent T315I from occurring, removing the most troublesome resistance complication from the set of potential treatment outcomes.

**Table 1:** $\widetilde {\text {IC}}_{50}$ (IC_50_ of mutant relative to wildtype, Eq. 4) values of Bcr-Abl1 mutants

Mutant ^a^	Imatinib	Nilotinib	Dasatinib	Bosutinib	Ponatinib	Axitinib ^b^	Asciminib
P223S	0.70	0.82	0.83	0.72	0.86		24.62
G250H	0.85	1.06	0.98	0.72	0.94		1.21
Q252H	2.78	2.41	1.44	0.98	5.56	1.92	17.96
Y253H	15.95	34.89	1.84	0.91	5.28	1.31	2.81
E255K	12.51	13.99	7.53	4.07	11.82		3.86
E255V	10.98	27.60	3.76	2.65	11.28	1.25	1.92
K294E	0.48	0.68	0.48	0.55	0.67		29.99
V299L	1.83	1.81	10.33	13.65	1.22	0.32	10.07
T315I	27.70	266.76	806.32	17.07	5.59	0.16	12.56
A337V	0.91	1.04	0.98	0.98	0.95		744.64
E355G	2.56	1.37	0.71	0.63	0.77		15.34
F359V	4.00	7.58	1.45	1.04	4.70	0.69	18.98
E459K	2.22	2.62	0.82	0.68	2.01		4.94
P465S	1.02	0.94	0.82	0.79	0.98		605.96
V468F	0.72	0.68	0.48	0.74	0.42		529.32
I502L	0.62	0.76	0.70	0.67	0.78		49.72

$\widetilde {\text {IC}}_{50}> 1$ indicates a mutation that is resistant to some degree. Note that drugs can sometimes effectively suppress weakly resistant mutations. Given values are the geometric mean of the $\widetilde {\text {IC}}_{50}$ from all sources that state a precise value (i.e. not an approximation, lower, or upper bound) for the given mutation [9–11, 15, 33–44]. The specific sources associated with each value are given in Additional file 2

^a^Mutant selection was based on limited data availability for asciminib. Mutations at underlined residues are only associated with asciminib resistance (preclinical data)

^b^Data for axitinib is only known for a limited subset of mutations but it is considered to be ineffective to native Abl1 and all resistance mutants except T315I and possibly V299L and F359V

Treatment with multiple drugs without overlapping resistance mechanisms is advantageous (at least in theory), as a single cell would have to become resistant to all drugs at once [12]. A recent development towards this aim is the design of allosteric inhibitors that target the myristoyl pocket of Bcr-Abl1. These have been in development for a long time (GNF-2, GNF-5) [13]. More recently, another drug candidate, asciminib (ABL001) [14], has been developed and is used in ongoing trials. Another potential advantage of combination therapy lies in an effective dose reduction for a synergistic combination of drugs. For a treatment to be effective, we need to slow down the growth of cancer cells. Combining current ATP-pocket binding TKIs is not very effective since they bind to the same site and effectively compete with one another. On the other hand, a combination of asciminib and an ATP-pocket TKI is more likely to be efficient since they do not compete in the same manner. Indeed, combinations of asciminib and nilotinib [15] as well as asciminib and ponatinib [16] have been shown to prolong survival in mouse xenograft models. In particular, nilotinib and asciminib alone resulted in mutation–based resistance, whereas the combination created a durable response [15].

A potential risk with drug combinations is that they can be more vulnerable to resistance under certain conditions. Generally, drug combinations reduce the risk of resistance as it is unlikely that any cell will adapt to both drugs at once [17]. However, this view has been challenged by a study showing that bacteria adapt quicker to synergistic drugs (i.e., a combination where the effect is greater than the sum of it’s parts) [18]. Such an effect may be present with allosteric inhibitors.

We have previously examined a drug rotation protocol, where a patient is moved between drugs of different resistant profiles before resistance is observed and showed that such a protocol can delay the onset of resistance [19]. A drug combination may prevent some of the issues associated with a rotation protocol, for instance selecting for compound mutations [20]. It is worth mentioning however, that there are algorithms for selecting combinations that minimise cross resistance [17]. Given that Bcr-Abl1 activity is in most cases crucial to CML cells, mutation-induced variations in the function of Bcr-Abl1 affect how prevalent these mutations are in practice [5, 21].

Modelling and simulations are widely used in cancer research [22–24]. When it comes to drug combinations, one method is to examine the signalling network to identify other candidate targets which might compensate for and lower the effect of inhibiting a primary target [6, 7, 25, 26]. In EGFR driven non small cell lung cancer, carefully timed sequential use of targeted therapies with cytotoxic chemotherapy can yield better effects [27] whereas in targeted therapy combinations there is a complex interplay of drug interaction and pharmacokinetics which must be accounted for [28]. In Philadelphia-chromosome positive acute lymphoblastic leukaemia, which has the same driver as CML, it may be possible to add radiation to targeted therapy for an improved effect [29].

The development of asciminib and discovery that axitinib inhibits the T315I mutant of Bcr-Abl1 provide opportunities for combination therapy that would be effective both in reducing tumour burden and in postponing resistance. This would however require tailoring of the drug-treatment schedule and examination of the efficacy of the therapy. To this end, we developed a computational protocol to study the effect of drug combinations subject to different timing of the drugs and based on their affinity to the drug target. Here, we examined: (I) Whether supplementation of standard TKIs by axitinib can suppress the development of T315I-based resistance; which was performed by calculating the growth rates of T315I under such combinations given known drug pharmacokinetics, and (II) Given that asciminib does not compete with current (ATP-pocket) drugs, how much can the total drug burden be lowered for a drug combination? Optimal doses and administration timings were predicted by numerically evaluating the inhibition over a range of drug doses. Then, for given optimal doses and timings, we predicted how sensitive the combination should be to resistance mutations.

## Methods

CML is commonly treated with a single TKI agent that targets Bcr-Abl1 (primarily imatinib, dasatinib and nilotinib, that vary in their resistance and side-effect profile). Under the influence of a single inhibitor at concentration *C*, the fractional growth rate *f*_*v*_ of a cell is

1 $$ f_{v} = \frac{1}{1 + \left(\frac{C}{IC_{50}} \right)^{m} }   $$

where IC_50_ is the concentration required for a 50% reduction in growth rate and *m* is the hill-coefficient which describes the sigmoidicity of the relationship. *m*=1 if the enzymatic reaction follows Michaelis Menten kinetics in the presence of the inhibitor. In practice this is not always the case and a range of values may be considered. Two cases need to be considered when two inhibitors are combined, namely (I) that their effect is exclusive and only one inhibitor can work at once, and (II) that their effect is nonexclusive and they act entirely independently [30, 31]. Other variants of interaction have effects which fall somewhere in between these two. In the exclusive case, Eq. 1 becomes

2 $$ f_{v} = \frac{1}{1 + \left(\frac{C_{1}}{(IC_{50})_{1}} + \frac{C_{2}}{(IC_{50})_{2}} \right)^{m}}   $$

and in the nonexclusive case:

3 $$ f_{v} = \frac{1}{1 + \left(\frac{C_{1}}{(IC_{50})_{1}} + \frac{C_{2}}{(IC_{50})_{2}} + \frac{C_{1}C_{2}}{(IC_{50})_{1}(IC_{50})_{2}} \right)^{m}}.   $$

IC_50_ values for mutations are known to vary somewhat between studies, depending on the experimental conditions [32]. Considering Bcr-Abl1 inhibitors, these values are most often measured in murine Ba/F3-cells transfected with a (mutated) form of Bcr-Abl1. To overcome a potential bias due to experimental uncertainties, we considered a number of studies where IC_50_ values were reported [9–11, 15, 33–44] (see Table 1 for the data used in this study specifically and Additional file 2 for the complete dataset). In part, the discrepancies between studies are variations in scale, where the relative sensitivity of mutations is consistent. Therefore, we used values relative to cells that carry the native sequence Bcr-Abl1 (Eq. 4), and in much of the analysis.

4 $$ \widetilde{\text{IC}}_{50} = \frac{\text{IC}_{50}^{(\text{mutant})}}{\text{IC}_{50}^{(\text{wildtype})}}   $$

Using $\widetilde {\text {IC}}_{50}$ enables us to concentrate on drug resistance rather then on the actual concentration of the drug that inhibits cellular growth in a given experiment.

### Effect normalisation and pharmacokinetics

The integral of *f*_*v*_ (Eqs. 1–3) over some period of time from *t*_0_ to *t*_1_

5 $$ \int_{t_{0}}^{t_{1}} f_{v}(t) dt   $$

can be used as a measurement of how effective a treatment is at inhibiting growth. If *f*_*v*_(*t*) is periodic, i.e. there exists a certain period *τ*=*t*_1_−*t*_0_ for which *f*_*v*_(*t*)=*f*_*v*_(*t*+*τ*), it can be shown that if the integrals of *f*_*v*_ over time (Eq. 5) are equal for two different treatments, their effects are also equal (Additional file 1). Note that at steady state the TKI blood plasma concentration profile, and by extension *f*_*v*_(*t*), is periodic with period *τ* – the dosing interval. The steady state concentration profile is given by [45]:

6 $$ {\begin{aligned} C(t) = \frac{SFDk_{a}}{V_{d}(k_{a} - k_{e})} \times \left[ \frac{exp(-k_{e} t)}{1 - exp(-k_{e} \tau)} - \frac{exp(-k_{a} t)}{1 - exp(-k_{a} \tau)} \right]. \end{aligned}}  $$

The dose, fractional bioavailability, salt factor and volume of distribution (*D*, *F*, *S*, *V*_*d*_ respectively) serve to determine the magnitude of the plasma concentration, while the absorption and elimination rates (*k*_*a*_, *k*_*e*_) and the dosing interval *τ* determine the shape of the concentration curve over time. The parameters were determined numerically to reproduce half-times and peak plasma concentrations from clinical studies given dosages and dosing intervals (vide infra).

A combination of drugs can lead to a lower total amount of drugs required while maintaining a treatment effect comparable to either drug alone. For two drugs normalised to the same effectiveness we can define a dose reduction factor *ζ* of a combination of *x* parts drug A and (1−*x*) parts drug B as (for an exclusive interaction)

7 $$ \begin{aligned} &\int_{0}^{\tau} \frac{1}{1 + \left(\frac{C_{A}(t)}{(IC_{50})_{A}} \right)^{m}} dt = \int_{0}^{\tau} \frac{1}{1 + \left(\frac{C_{B}(t)}{(IC_{50})_{B}} \right)^{m}} dt \\&= \int_{0}^{\tau} \frac{1}{1 + \left(\frac{x \zeta_{e} C_{A}(t)}{(IC_{50})_{A}} + \frac{(1 - x) \zeta_{e} C_{B}(t)}{(IC_{50})_{B}} \right)^{m}} dt. \end{aligned}   $$

For a nonexclusive interaction:

8 $$ {}{\begin{aligned} &\int_{0}^{\tau} \frac{1}{1 + \left(\frac{C_{A}(t)}{(IC_{50})_{A}} \right)^{m}} dt = \int_{0}^{\tau} \frac{1}{1 + \left(\frac{C_{B}(t)}{(IC_{50})_{B}} \right)^{m}} dt \\&= \int_{0}^{\tau} \frac{1}{1 + \left(\frac{x \zeta_{n} C_{A}(t)}{(IC_{50})_{A}} + \frac{(1-x) \zeta_{n} C_{B}(t)}{(IC_{50})_{B}} + \frac{x (1-x) \zeta_{n}^{2} C_{A}(t) C_{B}(t)}{(IC_{50})_{A} IC_{50})_{B}} \right)^{m}} dt. \end{aligned}}   $$

Note that if *ζ*=0.5 and *x*=0.5 (an equal amount of each drug, effect–wise) then the actual amount required of either drug is only 25% of what would be required of the respective drugs in a monodrug therapy.

It follows that we can calculate an effective IC_50_ value (IC$_{50}^{\text {eff}}$) for a drug combination (defined by the dose ratio *x*) in terms of a combined pseudo-concentration *C* by rearranging Eqs. 2 and 3 when *f*_*v*_=0.5 (exclusive interaction):

9 $$ \frac{1}{2} = \frac{1}{1 + \frac{Cx}{(IC_{50})_{1}} + \frac{C(1-x)}{(IC_{50})_{2}}}   $$

or (nonexclusive interaction)

10 $$ \frac{1}{2} = \frac{1}{1 + \frac{Cx}{(IC_{50})_{1}} + \frac{C(1-x)}{(IC_{50})_{2}} + \frac{C^{2}x(1-x)}{(IC_{50})_{1}(IC_{50})_{2}}}.   $$

Solutions to these equations are provided in the supplementary material, Eqs. S16 and S17.

**Fig. 1:**
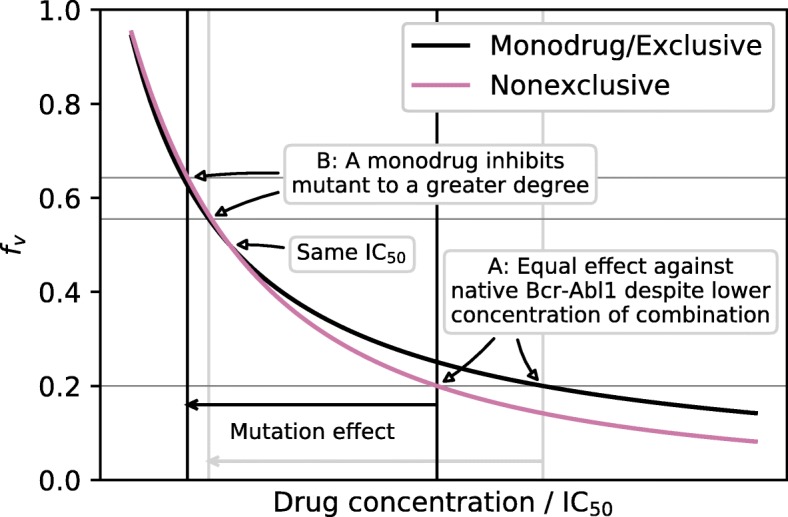
Dose-effect curves for a monodrug or exclusive combination and a nonexclusive combination (Eqs. 2 and 3). Given an effect target (*f*_*v*_=0.2 in the figure) the nonexclusive combination achieves it with a lower total drug concentration (**a**). The increased IC_50_ of a mutation has the same effect as reducing the drug concentration (Eqs. 2 and 3). At this lowered concentration, the monodrug or exclusive combination achieves a greater degree of inhibition (i.e. lower *f*_*v*_, **b**)

The IC$_{50}^{\text {eff}}$ falls somewhere between the IC_50_ values of each of the two drugs, but there is a separate effect which can produce an extra drug sensitivity for combinations. If two drugs are used to approximately the same degree and are approximately equally sensitive to a mutation, their effect will be less than expected, due to a difference in the shape of the dose-response curve as illustrated in Fig. 1. A demonstration of this effect is available in the supplementary material, Fig. S1.

### Implementation

Pharmacokinetic parameters for axitinib, asciminib and all approved TKIs were approximated from the literature (Table 2) by fitting *k*_*a*_, *k*_*e*_ and *V*_*d*_*S*^−1^*F*^−1^ to reproduce the maximum plasma concentration, time until maximum concentration, and concentration half time at the given doses from the listed sources. Axitinib supplementation testing was modelled as follows:

– Using Eq. 6 and Table 2 the concentration of each drug was calculated at 240 equidistant points during one day.

**Table 2:** Pharmacokinetic parameters estimated from literature

		*k*_*a*_	*k*_*e*_	*D*^c^	*τ*^c^	*VdS*−1*F*^−1^^a^
Imatinib	[49]	20	0.86	810	1	0.24
Nilotinib	[50]	30	1.5	760	0.5	0.31
Dasatinib	[51]	21	5.2	200	1	1.3
Bosutinib	[52]	10	0.71	750	1	2.5
Ponatinib	[53]	11	0.76	84	1	0.50
Axitinib	[54]	23	4.1	13 ^b^	1	0.15
Asciminib	[55]	27	1.6	88 ^b^	1	0.076

^a^Calculated as one variable during parametrization

^b^Estimates of a tolerable dose. No standard doses have been established as these are not conventional treatments

^c^From standard treatment [56]
– At each of the points in time above, the fractional growth rate of native sequence Bcr-Abl1 and T315I mutant cells was evaluated for imatinib, nilotinib, dasatinib and bosutinib alone and for each of them in combination with axitinib using Eqs. 1 and 2, respectively. T315I $\widetilde {\text {IC}}_{50}$ values used are listed in Table 1.
– The ratio of the native sequence and T315I mutant growth rates was then evaluated for each point in time (Eq. 11), vide infra (“Results” section).
– This was repeated with 48 different timing offsets (i.e. every 30 minutes) with regards to the dosing, and the optimum was selected, either on a minimum *χ* or a minimum average *χ* as a criterion.

The dose reduction and $\widetilde {\text {IC}}_{50}^{\text {eff}}$ for asciminib combinations were modelled as follows:

– The doses of asciminib, imatinib, nilotinib, dasatinib, bosutinib and ponatinib were normalised individually such that the integral of the *f*_*v*_(*t*) curves (Eq. 1) was equal to a given effect target (in this case 0.1) using an L-BFGS minimization algorithm, by calculating the concentration and *f*_*v*_ profiles as above (using 1000 points) and integrating numerically.
– The dose reduction factors *ζ* which reproduced the same effect target were then calculated for each combination using Eqs. 7 and 8 and the same L-BFGS minimization algorithm at 21 equidistant drug dose rations (*x*∈[0,1]) and 24 different dose offsets (every 1h).
– Using the dose ratio and offset that produced the greatest dose reduction (i.e., the lowest *ζ*) the $\widetilde {\text {IC}}_{50}^{\text {eff}}$ value for each mutation was calculated using Eq. 10 and data from Table 1.
– Mutations which might have a greater effect due to curve-shape effects (Fig. 1) were identified by evaluating whether the inhibition of the combination against that mutant was smaller than either monodrug would be against that mutation, calculated with Eqs. 3 or 1 respectively.

### Experimental procedures

#### Cell culture and drugs

KCL-22 cells were a donation from Prof. Leif Stenke and were grown in RPMI 1640 medium (with GlutaMAX; Gibco) with 10% FBS (Gibco) and 1% Pen Strep (Gibco). Imatinib (SignalChem), dasatinib (SignalChem) and asciminib (MedChemTronica) were purchased and dissolved in DMSO (SigmaAldrich).

#### Synergy assay

10^4^ cells were seeded per well in a 96-well cell culture plate with a twofold dilution series of imatinib, dasatinib or asciminib in medium. Each drug was measured in triplicate. After 48h, cell proliferation was measured using an MTS-assay (CellTiter 96 Aqueous One Solution Cell Proliferation Assay, Promega) as described by the manufacturer. The procedure was then repeated with drug combinations imatinib + dasatinib, imatinib + asciminib and dasatinib + asciminib at concentration ratios of 6.31:0.0130, 6.31:0.0524 and 0.0130:0.0524 respectively.

### Statistical methods

IC_50_ values and hill coefficients were estimated with a four parameter logistic curve using the rethinking Stan interface (Fig. S8) [46, 47]. Combination index (CI) plots were calculated using the method described in [31].

## Results

Throughout the results we have chosen to present data assuming a Hill coefficient *m*=2. Hill coefficients vary a lot between mutations and drugs [48]; *m*=2 was chosen as a representative value for Ba/F3 cells, and it is also consistent with our measurements in KCL-22 cells. Unfortunately, there are no in vivo measurements on CML progenitor cells. Results obtained with *m*=0.5 and *m*=1 are presented in Additional file 1.

### A combination involving axitinib and another ATP-competitive inhibitor only marginally lowers the selective advantage of t315I

Since axitinib inhibits T315I, and given that rotation protocols with another T315I inhibitor (ponatinib) were shown to be useful [19] we wanted to examine whether it is possible to lower the selective advantage of the T315I mutant by the use of axitinib in addition to other drugs. T315I is evolutionarily favoured if, under a given treatment protocol, it grows faster than cells that carry native Bcr-Abl1 (wildtype). Thus, ratio of the growth rates, *χ* (Eq. 11), gives an idea of whether or not T315I poses a threat assuming that T315I and wildtype cells grow at the same rate without inhibitors. If *χ*>1, T315I cells grow faster than wildtype cells, and the ultimate goal of axitinib supplementation would be to have a *χ* consistently below 1, where

11 $$ \chi = \frac{f_{v}^{\mathrm{(T315I)}}}{f_{v}^{\mathrm{(wildtype)}}}.   $$

**Fig. 2:**
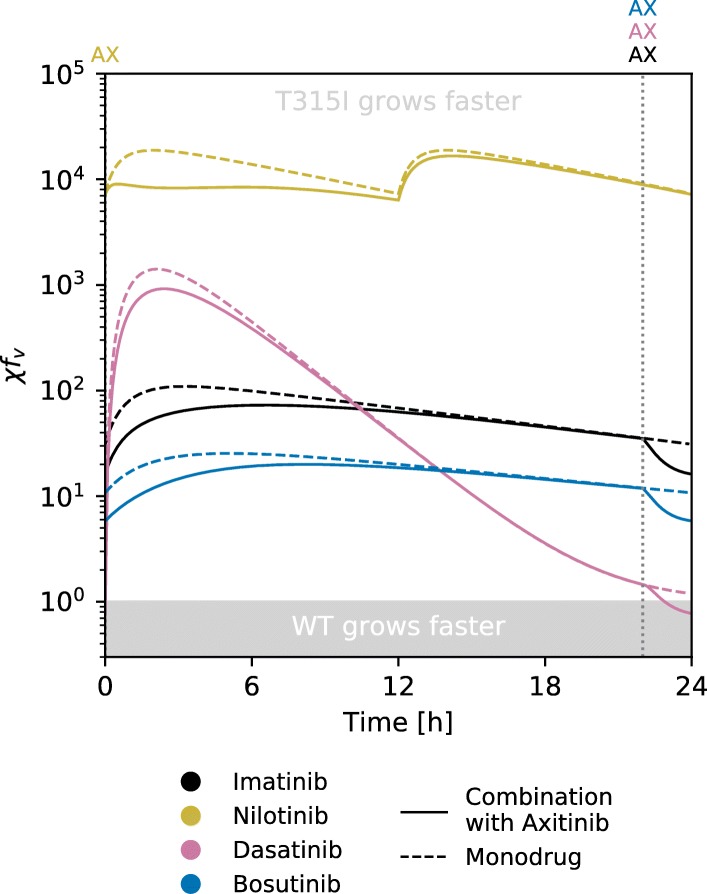
Simulations of the effects of axitinib supplementation in a standard TKI treatment. The standard TKI is taken at *t*=0 for imatinib, dasatinib and bosutinib, and at *t*=0 and *t*=12 for nilotinib. Axitinib administration is at *t*=0 when taken with nilotinib and at the time (*t*=22h) indicated by the vertical dotted lines when taken with each of the other drugs. Administration time for axitinib has been optimised to achieve the minimum instantaneous *χ*

Given how the concentrations of axitinib and the other drug vary during the day, we can find how quickly native Bcr-Abl1 containing cells and T315I cells are growing respectively using Eq. 2. We found that axitinib is not enough to yield *χ*<1 for any significant measure of time using drug concentrations based on the known level of drugs in the plasma (Fig. 2). A decrease in the rate of growth of cells that carry the T315I mutation can be achieved temporarily each day using an axitinib–dasatinib combination, since dasatinib is eliminated from the body rather quickly, but T315I-cells still carry a large growth rate advantage for most of the day. Giving axitinib about 2–3 h before the other TKI achieves the lowest instantaneous *χ* (Fig. 2) whereas giving axitinib in conjunction with, or slightly after the other TKI lowers T315I’s average advantage the most (Fig. S2).

As is expanded on further below, plasma concentrations are not necessarily a good match with inhibition effect in practice. Figs. S3 and S5 use effect-normalised doses. However while the axitinib’s effect is greater under these conditions, it is not enough to eliminate all advantage from T315I.

### Asciminib in combination with an ATP-pocket TKI allows for significant dose reduction

Asciminib is believed to be well tolerated enough that it can be given as a monodrug therapy (unlike axitinib which is not efficacious at tolerable doses). Nevertheless, as there is currently no evidence that asciminib is superior to current TKIs, there is good reason to attempt combination treatments. In principle a combination of several drugs without overlapping resistance mechanisms is most often beneficial from an evolution of resistance perspective, as the likelihood of one cell acquiring two different adaptations at once is lower than the odds of acquiring just one. Furthermore, because asciminib has a different binding site it is likely that its administration together with ATP-pocket TKIs could allow for dose reductions (Eqs. 3 and 8), this nonexclusive behaviour was consistent with our experiments. However, the combination may be overcome by resistance.

In practice, dose-activity relationships established mainly in Ba/F3 cells do not seem to mesh all that well with plasma concentration derived drug concentrations and clinical experience with resistance mutations in patients. In particular, when considering its IC_50_ against Abl1-transfected Ba/F3 cells, nilotinib seems more potent than experience would suggest. To remedy this we first normalise the doses of all drugs so that they are equally effective (under the measure specified in Eq. 5). This is justified by assuming that standard drug doses have been calibrated to minimise side effects while inhibiting proliferation enough to be an effective treatment. This normalisation is not intended as an actual change in doses, but rather as a rough model of all the other factors that might make up the difference between plasma concentration and the actual inhibition effect such as differences in drug transport into the cells and the degree of plasma protein binding.

Reproduction in the cancer stem cells, which are the only ones that can sustain resistance mutations over a long period of time, was calculated to be at most 1/7 of its normal rate under treatment to produce observed reductions of differentiated cells [57]. With 1/7 as an upper bound we set a rough estimate at a 1/10 (or 90%) reduction of the integral of *f*_*v*_ for an effective treatment. The drug concentrations needed to achieve this can be reached according to plasma concentrations [49–55] and Ba/F3-derived dose response curves.

**Fig. 3:**
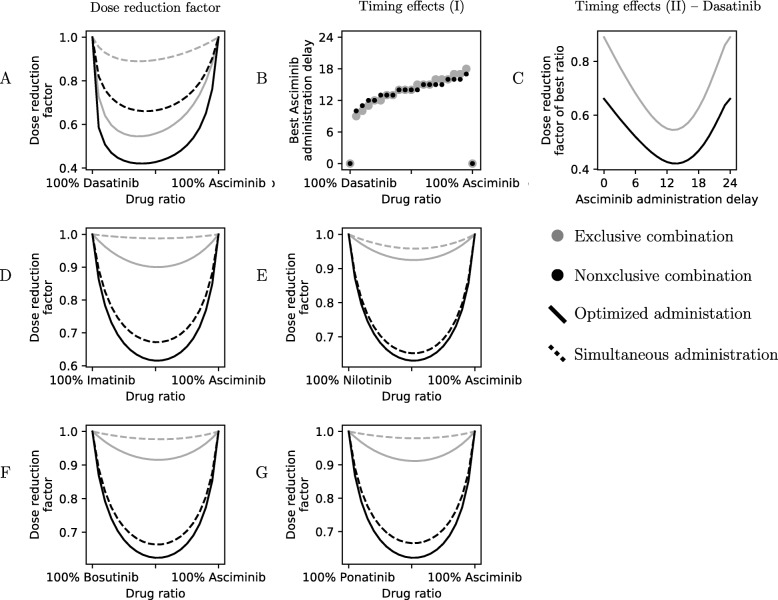
Predicted dose reduction of ATP-pocket TKI + asciminib combinations that are as effective as standard therapy. Grey lines shows effects assuming the combination has an exclusive interaction (Eq. 2) and black lines show a nonexclusive combination (Eq. 3). The latter is more likely for the combinations shown. Depending on whether the drugs are given simultaneously or in a staggered manner the effect changes. Dashed lines were calculated for a simultaneous administration whereas for the full lines the offset between taking one drug and the other was optimised to improve the dose reduction potential. **a**: A dasatinib-asciminib combination has a good dose reduction potential but it is sensitive to variations in administration timing and performs much better with a staggered administration schedule (**b**, **c**). **d**–**g**: Other combinations (imatinib, nilotinib, bosutinib and ponatinib, each combined with asciminib) perform similarly to one another, and are not very sensitive to administration timing (data not shown)

**Fig. 4:**
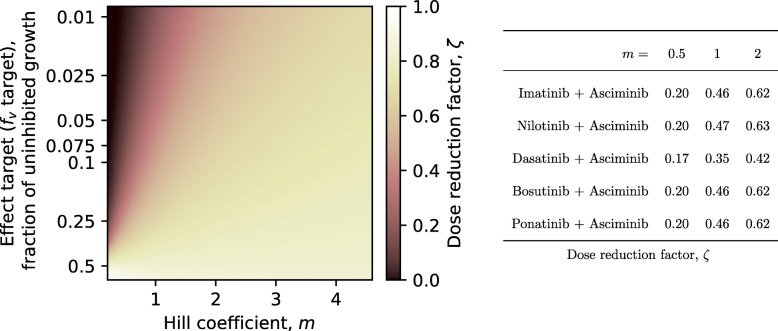
The dose reduction factor of a nonexclusive combination was calculated for a hypothetical drug combination with constant drug concentration profiles (*C*(*t*) for either drug). The best achievable dose reduction factor was calculated for five different drug combinations using a reasonable effect target of 0.1 and is shown on the right–hand side of the figure

Under these assumptions, in every case (Fig. 3), an even split between an ATP-pocket TKI and asciminib, at 25% the standard dose of each, taken 12 h apart is near optimal. In case that the hill coefficient *m*<1 the dose reduction potential increases, and if *m*>1 it is decreased (Fig. 4). Similarly, the choice of effect target affects the dose reduction potential. Neither the ratio nor the timing is particularly sensitive to variations (data not shown), so inevitable inaccuracies in precisely reproducing this in practice should only have minor effects on the results. The one exception is in a combination of dasatinib and asciminib (Fig. 3a) where, because of the rapid elimination of dasatinib from the body, there is a fairly significant difference between simultaneous and interleaved administration of the drugs, where the latter is preferable. For other combinations, simultaneous administration of the ATP-pocket TKI and asciminib seems to achieve nearly the same effect as optimally timed administration according to our modelling.

**Fig. 5:**
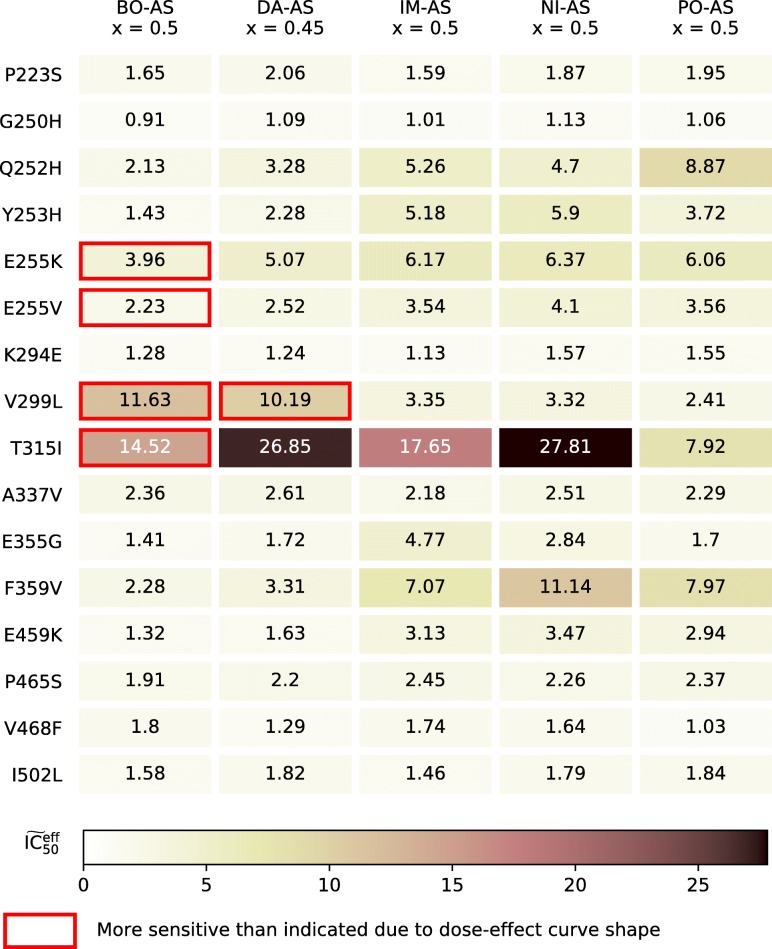
$\widetilde {\text {IC}}_{50}^{\text {eff}}$ (i.e. IC$_{50}^{\text {eff}}$ relative to native Bcr-Abl IC$_{50}^{\text {eff}}$) for selected mutations towards an asciminib + ATP-pocket TKI combination. Boxes highlighted in red are more sensitive towards mutations than the $\widetilde {\text {IC}}_{50}^{\text {eff}}$ would suggest (as described in Fig. 1) assuming a 90% inhibition target. Drug names are abbreviated as: imatinib – IM, nilotinib – NI, dasatinib – DA, bosutinib – BO, ponatinib – PO, and asciminib – AS

Using the optimised dose ratios from Fig. 3 we calculated the expected mutation sensitivity of the combinations assuming a nonexclusive interaction between ATP-pocket TKIs and asciminib according to Eq. 10 (Fig. 5). Most notably T315I remains an issue, although dasatinib–asciminib has a greatly reduced value compared to dasatinib alone. There are also some cases, mainly: V299L for dasatinib–asciminib, and V299L and T315I for bosutinib–asciminib, where a high $\widetilde {\text {IC}}_{50}$ and a dose-effect curve shape enhanced effect (Fig. 1 and S1) coincide, which may render them potent causes of resistance. Ponatinib–asciminib is not particularly vulnerable to any of the mutations considered (Fig. 5), and the dose reduction from the combination means ponatinib side-effects might become rare enough to make it viable as more than a last resort for T315I-positive patients; reduced doses of ponatinib have been shown to lower side effects [58]. Interestingly, our prediction is corroborated by recent results (published when this article was being revised) [16].

### Asciminib has synergistic interactions with ATP-pocket TKI in KCL-22 cells

**Fig. 6:**
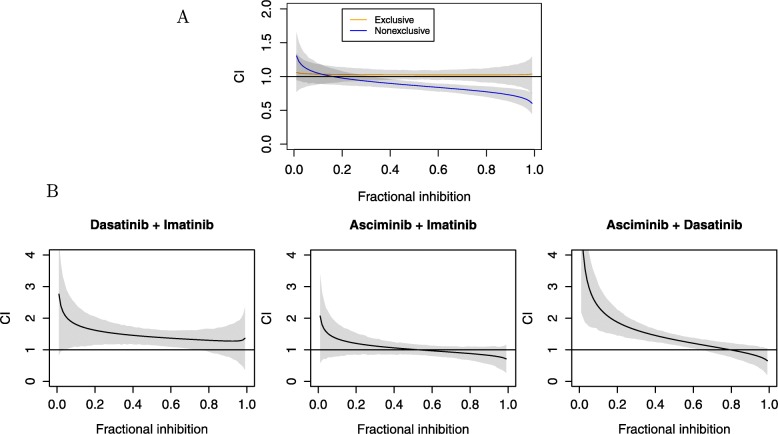
Combination index plots showing drug interactions in KCL-22 cells. Fractional inhibition is the complement to fractional velocity (i.e. 1 - *f*_*v*_), thus higher fractional inhibition is equivalent to slower growth. CI >1 indicates antagonism, whereas CI < indicates synergy. The Shaded grey regions show 89% compatibility intervals around the mean. **a**: Simulated perfectly exclusive and nonexclusive drug combination. **b**: KCL-22 cells not previously subjected to treatment. The results reveal that the combination of asciminib and dasatinib is not entirely non-exclusive (compare to panel **a**)

We compared the effects of an imatinib–dasatinib combination, both of them ATP-pocket inhibitors, with imatinib–asciminib and dasatinib–asciminib combinations (Fig. S8) to examine our assumption that the latter behaves as a nonexclusive combination. The combination of imatinib and dasatinib was always antagonistic, whereas the asciminib combinations exhibit synergistic effects at higher degrees of inhibition (Fig. 6b). This is consistent with the behaviour of a nonexclusive combination, as the additional interaction term grows in significance with the degree of inhibition (Eq. 3 and Fig. 6a). All combinations did however show a slight antagonistic bias compared to simulated interactions (Fig. 6), and although the reason for this is unknown the asciminib combinations nevertheless overcame it and demonstrated synergy at high degrees of inhibition. However, it implies that our modelling may overestimate the amount of dose reduction that might be possible.

### Using axitinib to supplement asciminib alone and in combination

It is not currently known whether asciminib alone can effectively treat T315I mutant CML, although its moderate T315I IC_50_ suggests it could be possible. In the eventuality that it cannot, it might be advantageous to add low dose axitinib. Another possibility is to add axitinib to one of the asciminib combinations discussed previously. Whereas axitinib had but a meagre effect with an ATP-pocket TKI (Fig. 2), it may benefit from the nonexclusive interaction with asciminib, and the lower T315I IC_50_ of it or its combinations making the evolutionary advantage easier to reverse.

We repeated the calculations from the axitinib combinations for an axitinib–asciminib and an axitinib–asciminib–bosutinib combination with two main modifications. asciminib was assumed to interact nonexclusively, which means growth rates were calculated using Eq. 3, and using a triple drug variant (see [30]). As detailed above, asciminib usage is not established, so we used normalised doses as described. Since axitinib is known to be ineffective against unmutated cells, it was normalised to yield a 5% growth rate reduction, which is close to what its plasma concentration would suggest.

**Fig. 7:**
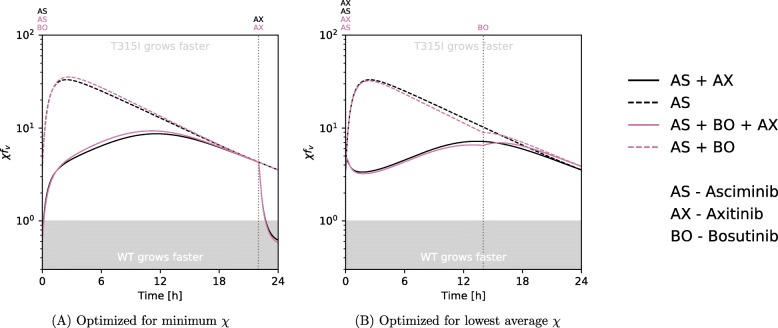
The effects of added axitinib to supplement a bosutinib and asciminib combination. Drug doses are normalised such that bosutinib–asciminib achieves 90% growth reduction together and axitinib achieves 5% growth reduction. Bosutinib–asciminib (left) and axitinib–asciminib (right) are administered at *t*=0. Axitinib (left) and bosutinib (right) administration is indicated by the dotted vertical lines. All timings were freely optimised; simultaneous administration is optimal when indicated

As can be seen in Fig. 7 axitinib has a greater effect under these conditions (see also Figs. S6 and S7 for other values of *m*). Compared to Figs. S3 and S5 which use the same effect normalisation, the difference in T315I advantage owing to the addition of axitinib is more noticeable. It does not eliminate the advantage of T315I entirely however.

## Discussion

We have evaluated the potential benefits of adding axitinib or asciminib to a standard drug treatment, under some basic assumptions about their interaction with available drugs. Supplementing any of the Abl1 inhibitors currently in use in CML by axitinib was not shown to achieve the ultimate goal of preventing T315I from emerging, but could slightly lower the odds for this. Given that each of these drugs is also associated with side effects or toxicities, such a combination does not seem to be of clinical relevance. Asciminib on the other hand appears to be promising in a combination treatment, both because it might reduce total drug burden, and because it seems to complement the resistance profiles of available drugs reasonably well given what we know now. It is not perfect however; in particular, it might be worth paying attention to V299L for a dasatinib–asciminib combination, and V299L and T315I for a bosutinib–asciminib combination, as those cases have both a degree of cross resistant and a possibly further increased advantage based on the dose-effect curve shape (Fig. 1 and S1). Moreover the T315I mutation apparently confers some resistance and continues to be a threat even when treatment includes a combination of a standard TKI and asciminib. A potential benefit of a ponatinib–asciminib combination is that asciminib is a substrate of the ATP-binding-cassette transporter ABCG2 [59], whereas ponatinib is an ABCG2 inhibitor [60] which might in part counteract ABCG2 upregulation as a resistance mechanism.

When modelling the effects of the drugs here, the hill coefficient was considered to be constant. In reality, however, it may vary with mutations in Ba/F3 cells [48]. This type of variation is thought to be important in acquisition of resistance in HIV [61]. Hill coefficients, or dose-effect curve slopes are rarely reported, and hence could not be included in regards to axitinib or asciminib, which were the main foci of the study. Including different hill coefficients for different mutants is a possible extension of this study that can be performed once such measurements are reported. However, it should be noted that in this study, the conclusions were not affected by choosing a different Hill coefficient as shown in Fig. 4 and S2–S7.

## Conclusions

This study suggests that a combination of conventional TKIs with asciminib can be useful for delaying the onset of resistance mutations that are detrimental to targeted therapy in CML. Computer simulations have shown synergistic effects for asciminib and clinically available Abl1 inhibitors. Experiments in KCL-22 cells have supported this, but suggested that the benefit would be lower than expected owing to synergism. There seem to be some intrinsic limitation on the use of the two drugs at the same time. In the case of the pan Abl1-mutant inhibitor ponatinib, such a combination can be useful to alleviate some side effects by reducing the dose of ponatinib that is needed to inhibit tumour growth. Our simulations did not show beneficial effects for combinations of TKIs with axitinib.

## Supplementary information

**Additional file 1** Figure S1–S8 depicting simulation results under a wider range of circumstances, dose response curves and derivations of mathematical formulae.

**Additional file 2** Comma separated values (csv) file of the complete averaged $\widetilde {	ext {IC}}_{50}$ dataset.

## Data Availability

The datasets supporting the conclusions of this article are included within the article (and its additional files). Simulation code (and datasets) are available at github.com/Sandalmoth/combination-kinetics-public.
