# Supplementary material for: The effects of combination treatments on drug resistance in chronic myeloid leukaemia: an evaluation of the tyrosine kinase inhibitors axitinib and asciminib
Source: BMC Cancer. 2020 May 7;20:397. doi: 10.1186/s12885-020-06782-9 (PMC7204252; doi:10.1186/s12885-020-06782-9)
Supplement: Supplementary file 1 — Additional file 1 Figure S1–S8 depicting simulation results under a wider range of circumstances, dose response curves and derivations of mathematical formulae. [file 12885_2020_6782_MOESM1_ESM.pdf]

Derivations of mathematical formulae

Equal f_v integral implies equal effectiveness

Assume exponential growth with a variable growth rate

$$\frac{dN}{dt} = r(t)N \tag{S1}$$

which has the solution

$$N(t) = N_0 \exp\left[\int_0^t r(\xi)d\xi\right] \tag{S2}$$

Let $r(t)$ have period τ s.t. $t = n\tau + \epsilon$ where $n \in [0, 1, 2, \dots]$ and $0 \leq \epsilon < \tau$, then

$$\int_0^t r(\xi)d\xi = n\int_0^\tau r(\xi)d\xi + \int_0^\epsilon r(\xi)d\xi. \tag{S3}$$

Thus for two different rate functions $r_1(t)$ and $r_2(t)$, with the same period, whenever $\epsilon = 0$ that have been normalised such that

$$\int_0^\tau r_1(\xi) = \int_0^\tau r_2(\xi). \tag{S4}$$

We get

$$\int_0^t r_1(\xi)d\xi = n\int_0^\tau r_1(\xi)d\xi = n\int_0^\tau r_2(\xi)d\xi = \int_0^t r_2(\xi)d\xi \tag{S5}$$

and their growth curves (S2) will thus follow the same long term trend

$$N(n\tau) = \exp\left[\int_0^{n\tau} r_1(\xi)d\xi\right] = \exp\left[\int_0^{n\tau} r_2(\xi)d\xi\right] \tag{S6}$$

In our application $r(t)$ is given by $f_v(t)$.

Drug combinations can be more vulnerable to resistance

For simplicity we, treat drug concentrations as constant, which implies that their effect, f_v, is also constant. This makes it simpler to compare treatments as we can safely ignore the integral (Equation S4). Equal f_v alone implies equal effect under this assumption. Consider the effects of a 50:50 drug combination, where both drugs have an equal unknown $\widetilde{\mathrm{IC}}_{50}$ (i.e. mutant IC_{50} divided by native Bcr-Abl1 IC_{50}) for a mutation.

The concentrations C_1 and C_{12} below are given as multiples of the IC_{50} . The equal effect against wildtype normalisation condition implies that

$$f_v = \frac{1}{1+C_1} = \frac{1}{1+C_{12}+\frac{1}{4}C_{12}^2}. \tag{S7}$$

From that it follows that the necessary concentration for the monodrug is

$$C_1 = \frac{1}{f_v} - 1 \tag{S8}$$

and the necessary total concentration for the combination

$$C_{12} = \frac{2}{\sqrt{f_v}} - 2. \tag{S9}$$

The combination is more vulnerable to resistance if the mutant grows faster at the drug concentrations specified by the above equation, i.e., if

$$\frac{1}{1+\frac{(\frac{1}{f_v}-1)}{\widetilde{\mathrm{IC}}_{50}}} < \frac{1}{1+\frac{\frac{1}{2}(\frac{2}{\sqrt{f_v}}-2)}{\widetilde{\mathrm{IC}}_{50}}+\frac{\frac{1}{2}(\frac{2}{\sqrt{f_v}}-2)}{IC_{50}}+\frac{\frac{1}{4}(\frac{2}{\sqrt{f_v}}-2)^2}{\widetilde{\mathrm{IC}}_{50}^2}} \tag{S10}$$

which means

$$\frac{(\frac{1}{f_v}-1)}{\widetilde{\mathrm{IC}}_{50}} > \frac{(\frac{2}{\sqrt{f_v}}-2)}{\widetilde{\mathrm{IC}}_{50}} + \frac{(\frac{2}{\sqrt{f_v}}-2)^2}{4\widetilde{\mathrm{IC}}_{50}^2} \tag{S11}$$

$$\frac{1}{f_v} - 1 > \frac{2}{\sqrt{f_v}} - 2 + \frac{(\frac{2}{\sqrt{f_v}}-2)^2}{4\widetilde{\mathrm{IC}}_{50}} \tag{S12}$$

$$1 + \frac{1}{f_v} > \frac{2}{\sqrt{f_v}} + \frac{\frac{1}{f_v} - \frac{2}{\sqrt{f_v}} + 1}{\widetilde{\mathrm{IC}}_{50}} \tag{S13}$$

$$\widetilde{\mathrm{IC}}_{50} > \frac{1+\frac{1}{f_v}-\frac{2}{\sqrt{f_v}}}{1+\frac{1}{f_v}-\frac{2}{\sqrt{f_v}}} \tag{S14}$$

$$\widetilde{\mathrm{IC}}_{50} > 1 \tag{S15}$$

In other words the drug combination is less effective against the mutation than a monodrug therapy assuming it is equally effective against the wildtype so long as $\widetilde{\mathrm{IC}}_{50} > 1$ (i.e., a resistant mutation). The effect is present so long as the mutant is approximately equally resistant towards both drugs, see Figure S1.

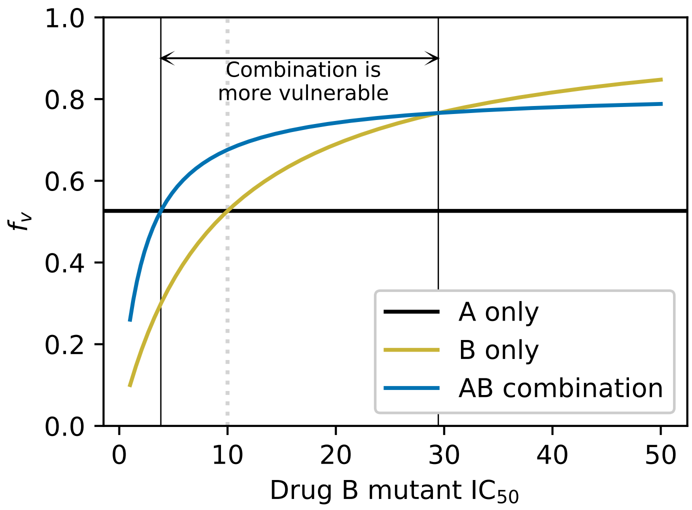

Figure S1: The doses of drug A and B were individually normalised to have the same effect on wildtype cells for each value of the drug B mutant IC_{50}. The growth rate of mutant cells is shown for each drug, calculated according to Equation 1 for A and B and Equation 3 for a drug combination AB, given the concentrations at each point provided by the effect normalisation against wildtype cells. Note the region where the combination has the highest mutant growth rate, implying a greater sensitivity towards the mutation.

Solutions for Equations 9 and 10

For the exclusive case (Equation 9), letting $a := (IC_{50})_1$ and $b := (IC_{50})_2$ for brevity:

$$C = \frac{1}{\frac{x}{a} + \frac{1-x}{b}} = \frac{ab}{a - ax + bx}. \tag{S16}$$

Similarly for the nonexclusive (Equation 10) case:

$$C = \frac{a - ax + bx + \sqrt{a^2(x-1)^2 - 6abx(x-1) + b^2x^2}}{2(x-1)x}. \tag{S17}$$

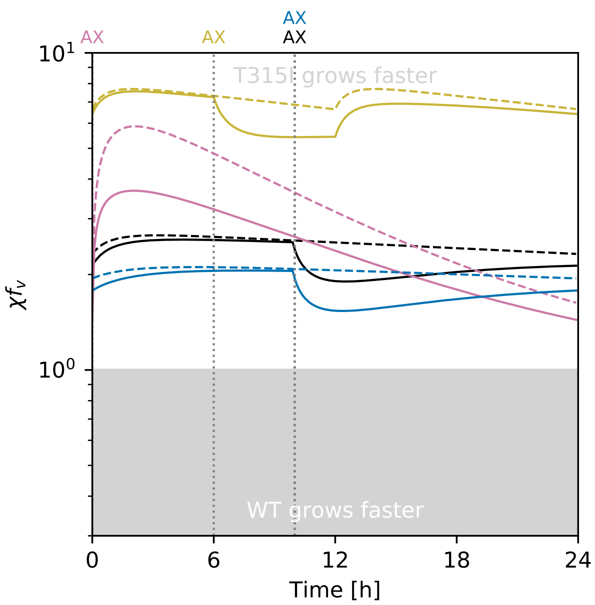

(A) $m = 0.5$

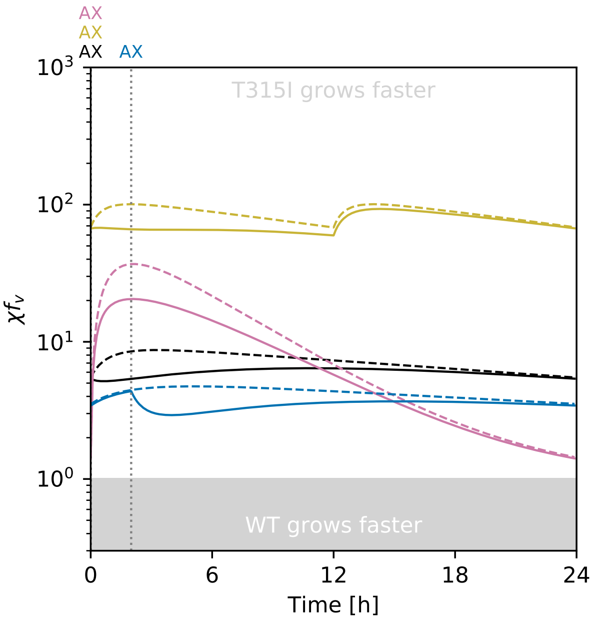

(B) $m = 1$

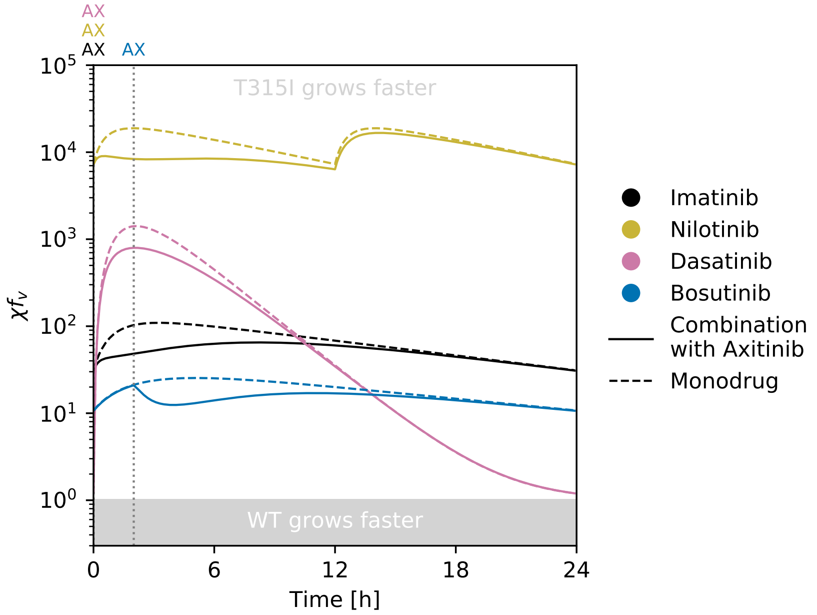

(C) $m = 2$

Figure S2: Simulations of the effects of axitinib supplementation in a standard TKI treatment. The standard TKI is taken at $t = 0$ for imatinib, dasatinib and bosutinib, and at $t = 0$ and $t = 12$ for nilotinib. Axitinib administration is indicated by the vertical dotted lines and has been optimised to achieve the lowest average χ.

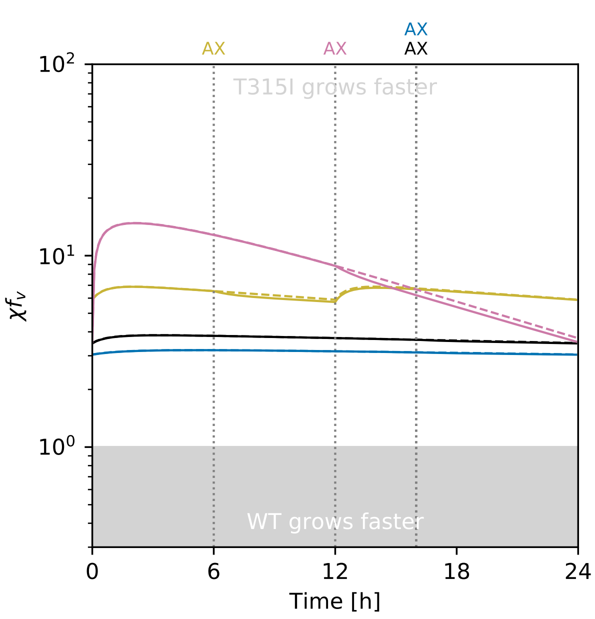

(A) $m = 0.5$

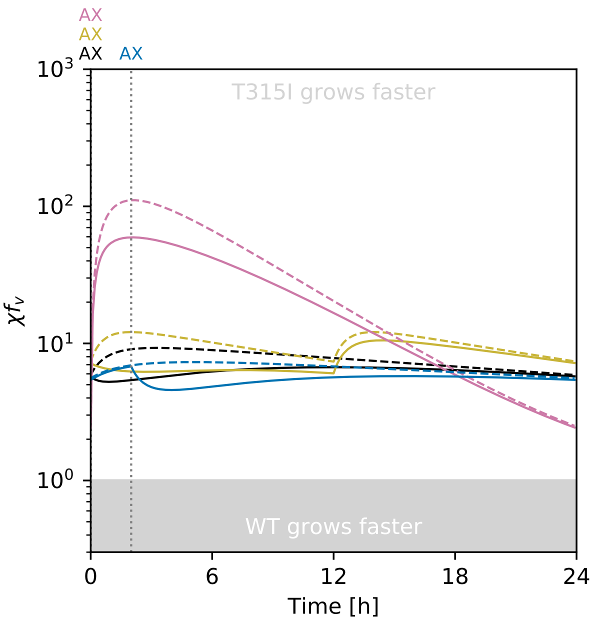

(B) $m = 1$

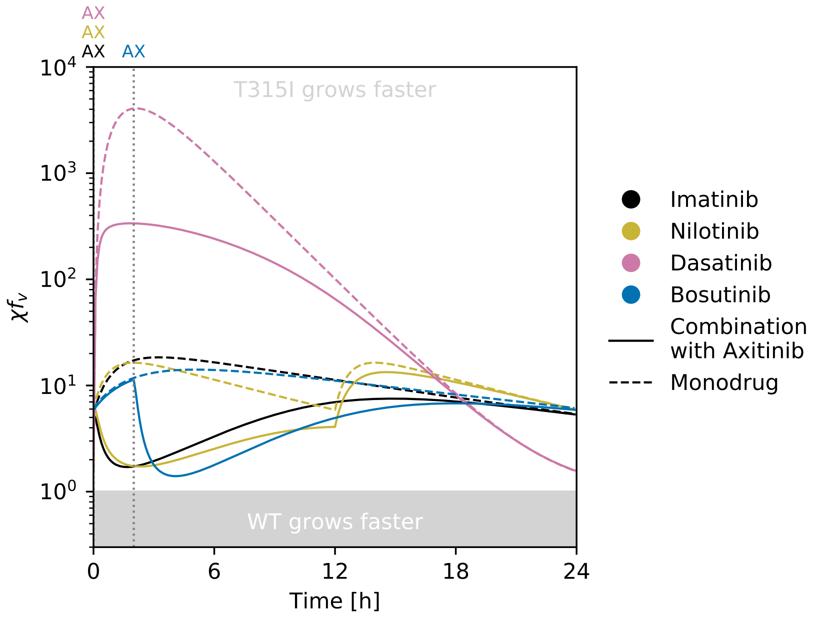

(C) $m = 2$

Figure S3: Simulations of the effects of axitinib supplementation in a standard TKI treatment. The standard TKI is taken at $t = 0$ for imatinib, dasatinib and bosutinib, and at $t = 0$ and $t = 12$ for nilotinib. Axitinib administration is indicated by the vertical dotted lines and has been optimised to achieve the lowest average χ. Treatment effects have been normalised such that imatinib, nilotinib, dasatinib and bosutinib cause a 90% growth reduction and axitinib causes a 5% growth reduction.

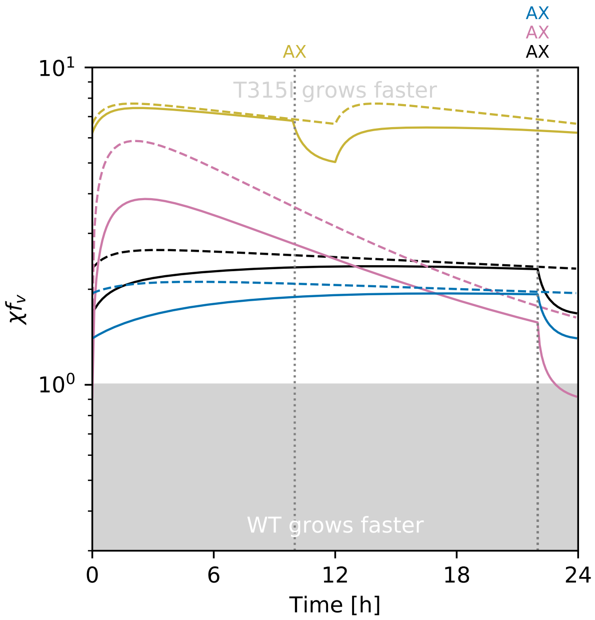

(A) $m = 0.5$

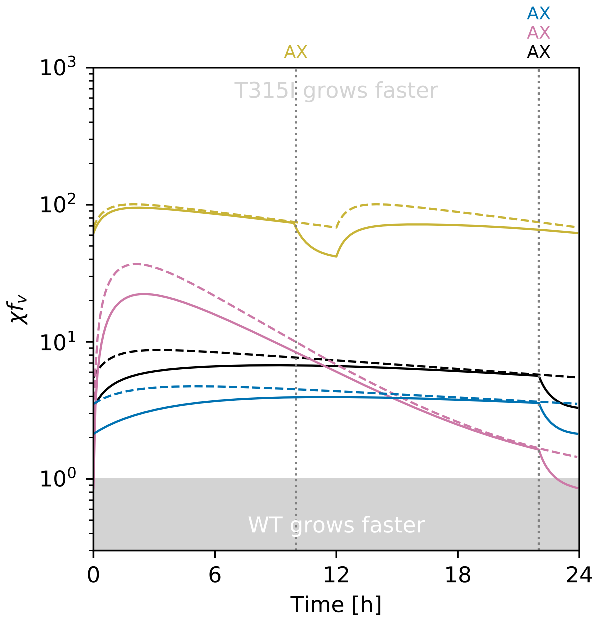

(B) $m = 1$

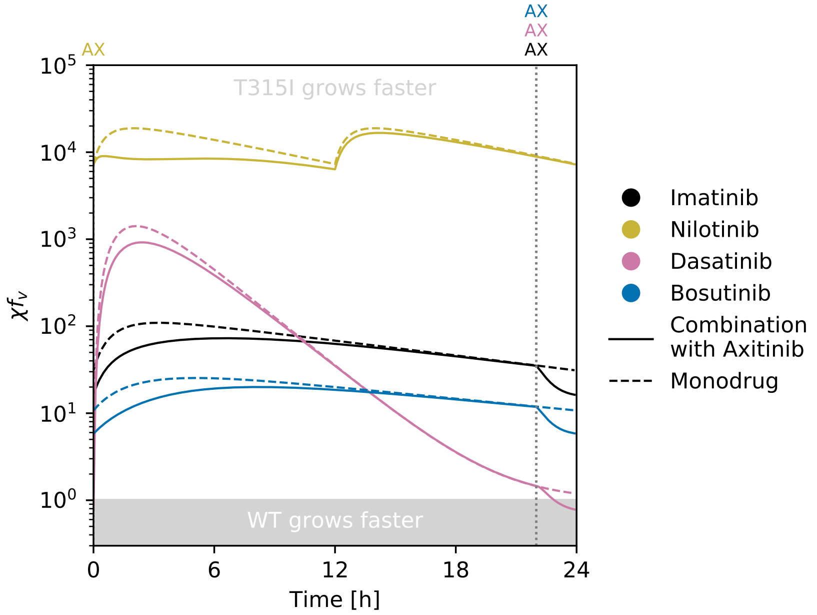

(C) $m = 2$

Figure S4: Simulations of the effects of axitinib supplementation in a standard TKI treatment. The standard TKI is taken at $t = 0$ for imatinib, dasatinib and bosutinib, and at $t = 0$ and $t = 12$ for nilotinib. Axitinib administration is indicated by the vertical dotted lines and has been optimised to achieve the instantaneous χ.

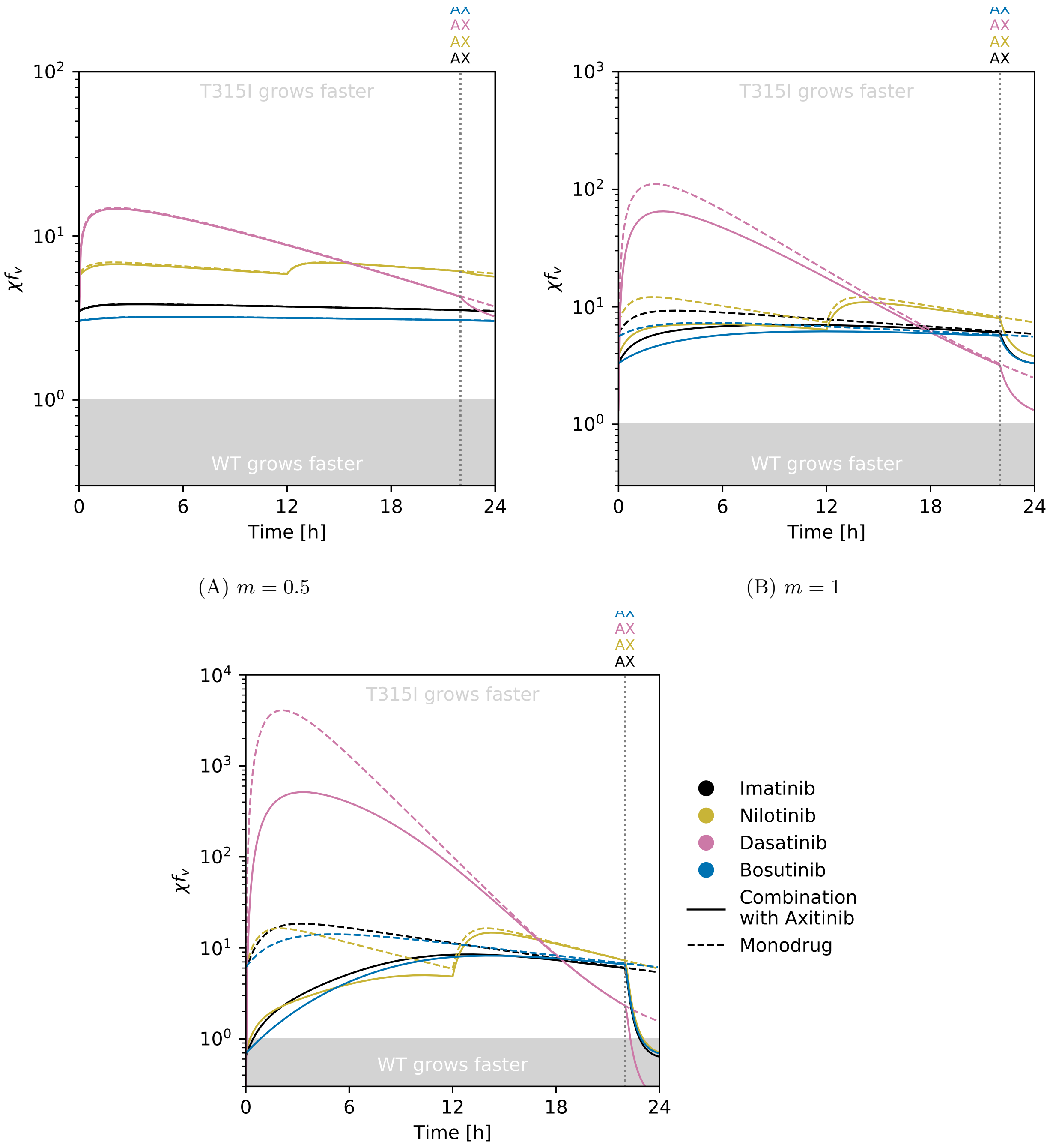

(A) $m = 0.5$

(B) $m = 1$

(C) $m = 2$

Figure S5: Simulations of the effects of axitinib supplementation in a standard TKI treatment. The standard TKI is taken at $t = 0$ for imatinib, dasatinib and bosutinib, and at $t = 0$ and $t = 12$ for nilotinib. Axitinib administration is indicated by the vertical dotted lines and has been optimised to achieve the lowest instantaneous χ. Treatment effects have been normalised such that imatinib, nilotinib, dasatinib and bosutinib cause a 90% growth reduction and axitinib causes a 5% growth reduction.

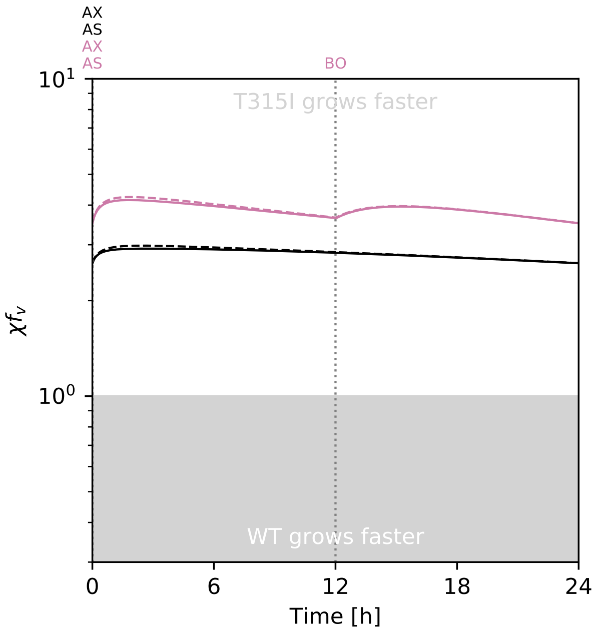

(A) $m = 0.5$

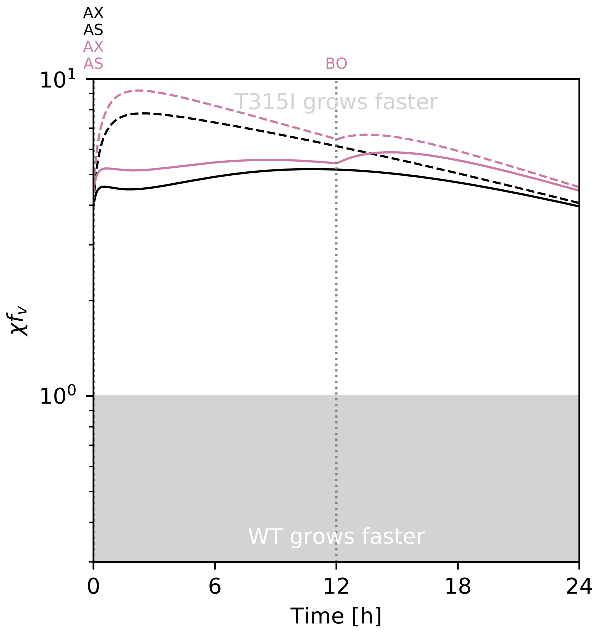

(B) $m = 1$

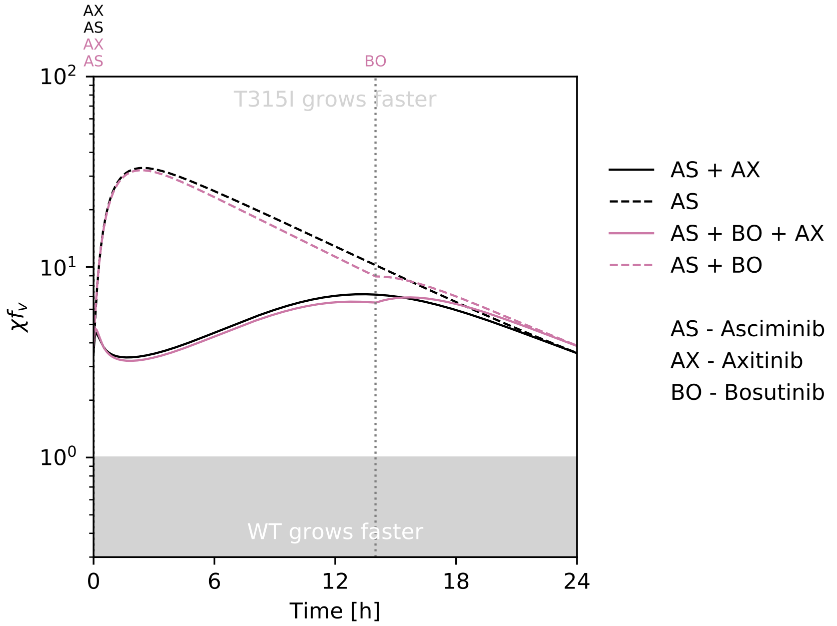

(C) $m = 2$

Figure S6: The effects of added axitinib to supplement a bosutinib and asciminib combination. Drug doses are normalised such that bosutinib–asciminib achieves 90% growth reduction together and axitinib achieves 5% growth reduction. Axitinib and asciminib (simultaneous is optimal) are administered at $t = 0$, bosutinib administration is indicated by the dotted vertical lines. All administration timings were optimised for minimum average χ.

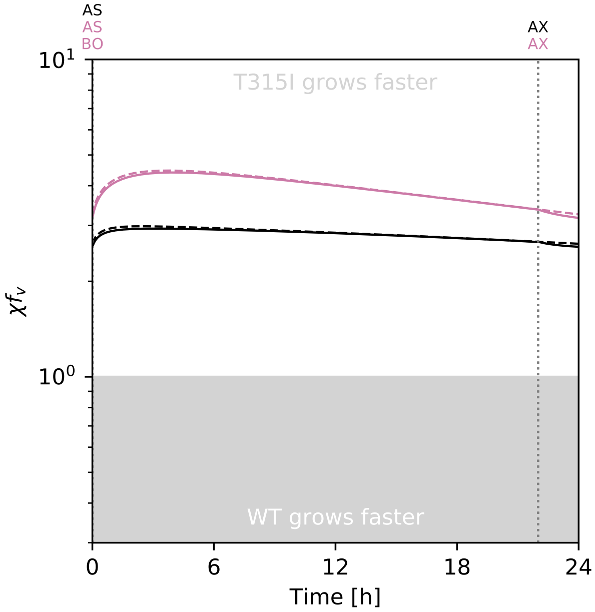

(A) $m = 0.5$

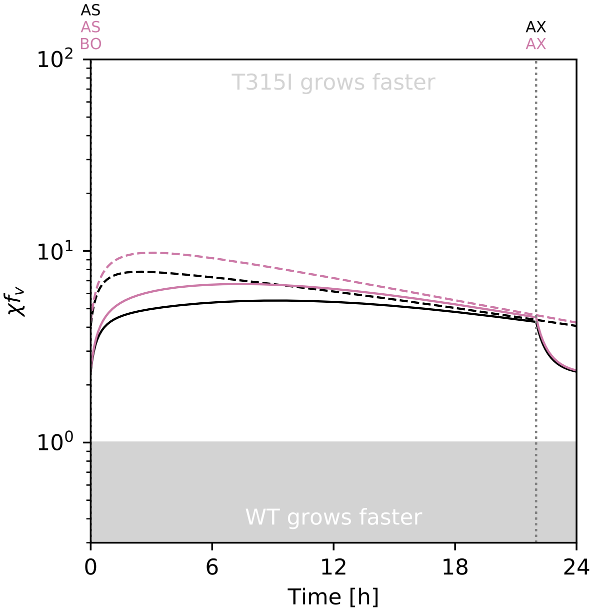

(B) $m = 1$

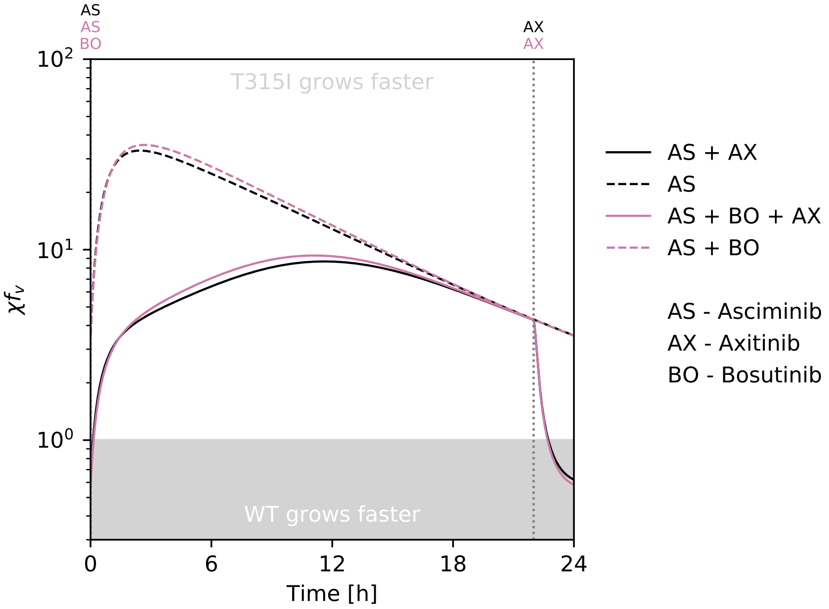

(C) $m = 2$

Figure S7: The effects of added axitinib to supplement a bosutinib and asciminib combination. Drug doses are normalised such that bosutinib–asciminib achieves 90% growth reduction together and axitinib achieves 5% growth reduction. Bosutinib and asciminib are administered at $t = 0$, axitinib administration is indicated by the dotted vertical lines. All administration timings were optimised for minimum instantaneous χ.

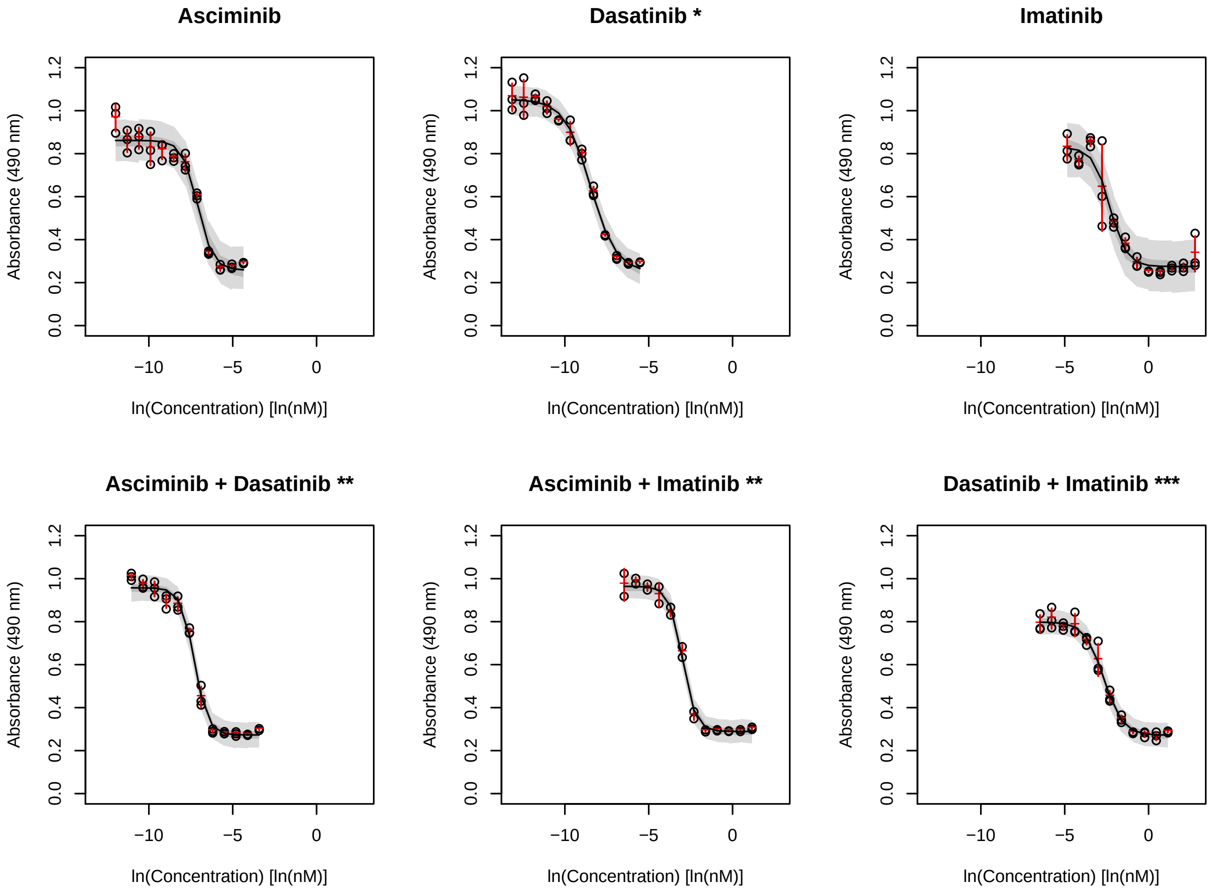

Figure S8: Dose response curves of KCL-22 cells cultured in the presence of a range of inhibitors. 490 nm absorbance using the Celltiter MTS assay correlates linearly with the number of viable cells. The red crosses indicate mean ± standard deviation of MTS assay results. The black line shows the mean, the light grey shaded region shows the 89% compatibility interval for posterior predictive simulations and the dark grey shaded region shows the 89% compatibility interval for the mean for the predicted dose response curve. Asterisks (*, ** and ***) indicate experiment batches, and incubation times varies slightly between them.
